# Supplementary material for: A novel approach for classifying monoamine neurotransmitters by applying machine learning on UV plasmonic-engineered auto fluorescence time decay series (AFTDS)
Source: Nanoscale Adv. 2025 Oct 14;7(24):8013–22. doi: 10.1039/d5na00416k (PMC12577017; doi:10.1039/d5na00416k)
Supplement: NA-007-D5NA00416K-s001 [file NA-007-D5NA00416K-s001.pdf]

Supplementary Information (SI) for Nanoscale Advances.
This journal is © The Royal Society of Chemistry 2025

Figure S1. Illustration of data indexing adjustment during preprocessing.

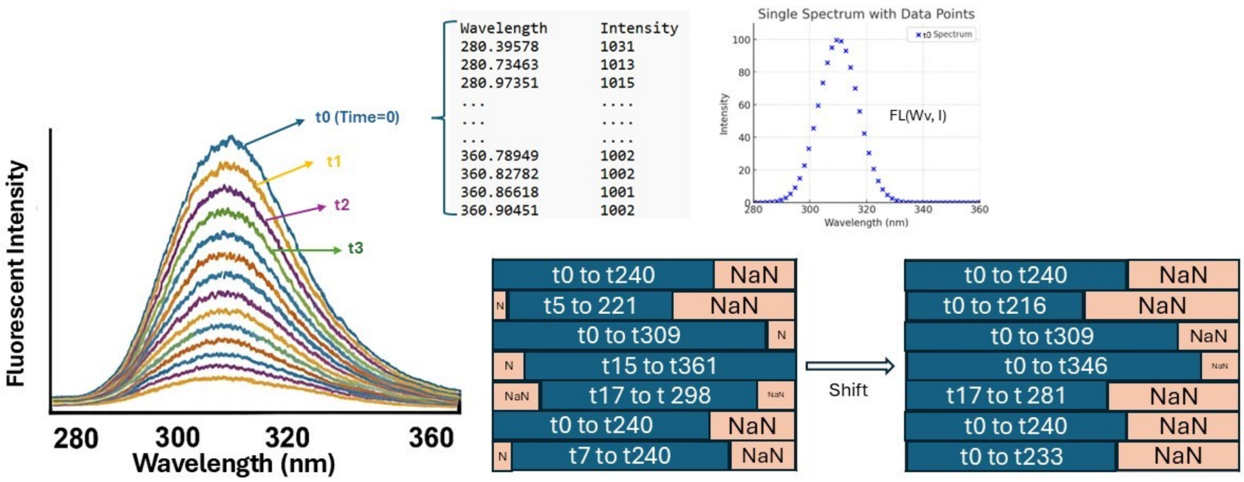

The AFTDS data was recorded as a series of spectra spanning 280–360 nm after sequential laser exposure over 2-3 minutes with 0.5 seconds as the exposure time. Each fluorescence time series originally contained 240–360 spectra (collected at 0.5 s intervals over 2–3 minutes). When saving autofluorescence data, the spectrometer software starts numbering each spectra from t0 when shutter opens. However, collection of actual spectra data might start at a later time with higher t number (e.g., t17) since adjustment of sample position might be necessary to find one spot with high fluorescence signal. The figure above illustrates this for clarification.

Before the data can be utilized effectively for machine learning models, it must first undergo curation to ensure it is appropriately structured. The goal of the task is to predict the molecule type based on the time series data of fluorescent intensities. Therefore, the dataset needs to be reshaped, where the time intervals are reflected in separate columns, representing the intensity values captured at different time points. Each row should correspond to the time dependent fluorescent intensity at a particular wavelength, with its name and spot number (Seg) serving as the label. This reshaped data structure is illustrated in Table S1.

Table S1. The dataset with time series values across different spots and molecules.

wavelength	Tag	Seg	t0	t1	t2	t3	t4	t5	t6	...	t352	t353	t354	t355	t356	t357	t358	t359	t360	t361
280.30017	DA 100 uM	S1	NaN	NaN	NaN	NaN	NaN	NaN	NaN	...	NaN	NaN	NaN	NaN	NaN	NaN	NaN	NaN	NaN	NaN
280.30017	DA 100 uM	S2	1026.0	1042.0	1043.0	1049.0	1039.0	1046.0	1046.0	...	NaN	NaN	NaN	NaN	NaN	NaN	NaN	NaN	NaN	NaN
280.30017	DA 100 uM	S3	1042.0	1036.0	1043.0	1043.0	1039.0	1038.0	1040.0	...	NaN	NaN	NaN	NaN	NaN	NaN	NaN	NaN	NaN	NaN
280.30017	DA 1000 uM	S1	1030.0	1046.0	1040.0	1046.0	1044.0	1046.0	1046.0	...	NaN	NaN	NaN	NaN	NaN	NaN	NaN	NaN	NaN	NaN
280.30017	DA 1000 uM	S2	1044.0	1048.0	1043.0	1045.0	1043.0	1048.0	1046.0	...	NaN	NaN	NaN	NaN	NaN	NaN	NaN	NaN	NaN	NaN
...	...	...	...	...	...	...	...	...	...	...	...	...	...	...	...	...	...	...	...	...
359.41248	NE 1000 uM	S2	1030.0	1044.0	1047.0	1041.0	1042.0	1050.0	1048.0	...	NaN	NaN	NaN	NaN	NaN	NaN	NaN	NaN	NaN	NaN
359.41248	NE 1000 uM	S3	NaN	NaN	NaN	NaN	NaN	NaN	NaN	...	NaN	NaN	NaN	NaN	NaN	NaN	NaN	NaN	NaN	NaN
359.41248	NE 500 uM	S1	NaN	1033.0	1027.0	1027.0	1031.0	1028.0	1029.0	...	NaN	NaN	NaN	NaN	NaN	NaN	NaN	NaN	NaN	NaN
359.41248	NE 500 uM	S2	NaN	NaN	NaN	NaN	NaN	NaN	NaN	...	NaN	NaN	NaN	NaN	NaN	NaN	NaN	NaN	NaN	NaN
359.41248	NE 500 uM	S3	NaN	NaN	1068.0	1073.0	1069.0	1074.0	1067.0	...	NaN	NaN	NaN	NaN	NaN	NaN	NaN	NaN	NaN	NaN

As depicted in Table S1, it can be observed that some rows do not begin at time zero. This results in missing values (Nan) at the start of certain rows. To address this issue, each row needs to be shifted left to eliminate the leading Nan values, effectively aligning all rows to start at the same initial time point. The corrected data are displayed in Table S2.

Table S2. The reshaped dataset with time series values across different spots and molecules.

Time →

Wavelength ↓

wavelength	Seg	Tag	t0	t1	t2	t3	t4	t5	t6	...	t8	t9	t10	t11	t12	t13	t14	t15	t16
280.30017	S1	DA	1044.0	1037.0	1041.0	1047.0	1046.0	1040.0	1042.0	...	1043.0	1039.0	1041.0	1042.0	1047.0	1052.0	1042.0	1048.0	1047.0
280.30017	S1	DA	1048.0	1049.0	1043.0	1043.0	1040.0	1041.0	1049.0	...	1041.0	1040.0	1041.0	1039.0	1042.0	1047.0	1043.0	1046.0	1043.0
280.30017	S1	DA	1040.0	1046.0	1040.0	1042.0	1038.0	1039.0	1045.0	...	1043.0	1048.0	1045.0	1041.0	1046.0	1046.0	1046.0	1045.0	1041.0
280.30017	S1	DA	1046.0	1041.0	1044.0	1044.0	1048.0	1047.0	1046.0	...	1050.0	1048.0	1043.0	1040.0	1048.0	1039.0	1044.0	1042.0	1048.0
280.30017	S1	DA	1046.0	1050.0	1044.0	1045.0	1043.0	1045.0	1043.0	...	1042.0	1044.0	1047.0	1047.0	1048.0	1045.0	1046.0	1053.0	1043.0
...	...	...	...	...	...	...	...	...	...	...	...	...	...	...	...	...	...	...	...
359.41248	S3	NE	1028.0	1028.0	1032.0	1033.0	1030.0	1032.0	1031.0	...	1032.0	1028.0	1026.0	1035.0	1028.0	1036.0	1034.0	1030.0	1026.0
359.41248	S3	NE	1034.0	1031.0	1029.0	1029.0	1025.0	1025.0	1031.0	...	1031.0	1031.0	1026.0	1030.0	1026.0	1032.0	1027.0	1027.0	1027.0
359.41248	S3	NE	1025.0	1024.0	1023.0	1021.0	1024.0	1023.0	1024.0	...	1025.0	1021.0	1026.0	1025.0	1027.0	1020.0	1031.0	1021.0	1025.0
359.41248	S3	NE	1025.0	1026.0	1025.0	1022.0	1021.0	1024.0	1026.0	...	1026.0	1027.0	1021.0	1023.0	1025.0	1022.0	1026.0	1024.0	1024.0
359.41248	S3	NE	1029.0	1027.0	1026.0	1027.0	1027.0	1027.0	1027.0	...	1027.0	1027.0	1027.0	1027.0	1027.0	1027.0	1027.0	1027.0	1027.0

Once the rows are adjusted to start from time zero, the next step is to compute the minimum and maximum lengths of the non-Nan values in each row. This is essential for understanding the variation in the recorded intensity values across time and ensuring consistency in data processing. It is assumed that the intensity values are dependent on each other through time, making the time series aspect crucial for accurate prediction of the molecule type. By aligning and trimming the time series data appropriately, the machine learning model will be better equipped to capture the underlying patterns in fluorescent intensity across different molecules.

To address the varying lengths of the time series data, it is necessary to feed fixed-length vectors into the LSTM model. Based on the minimum length of the time series, which is 17 (data from t0 to t16), any time series longer than this length will be sliced into smaller segments, each of length 17. This technique, referred to as slicing time series, augments the dataset by creating multiple fixed-length inputs from longer sequences. By ensuring that each segment conforms to the fixed input size (17), the model can better generalize and handle varying input lengths during training.

Figure S2. A) SEM image of the coffee ring pattern formed by the molecular solution, B) Borderline of the coffee ring pattern, and C) STEM image of an individual AlCNC particle.

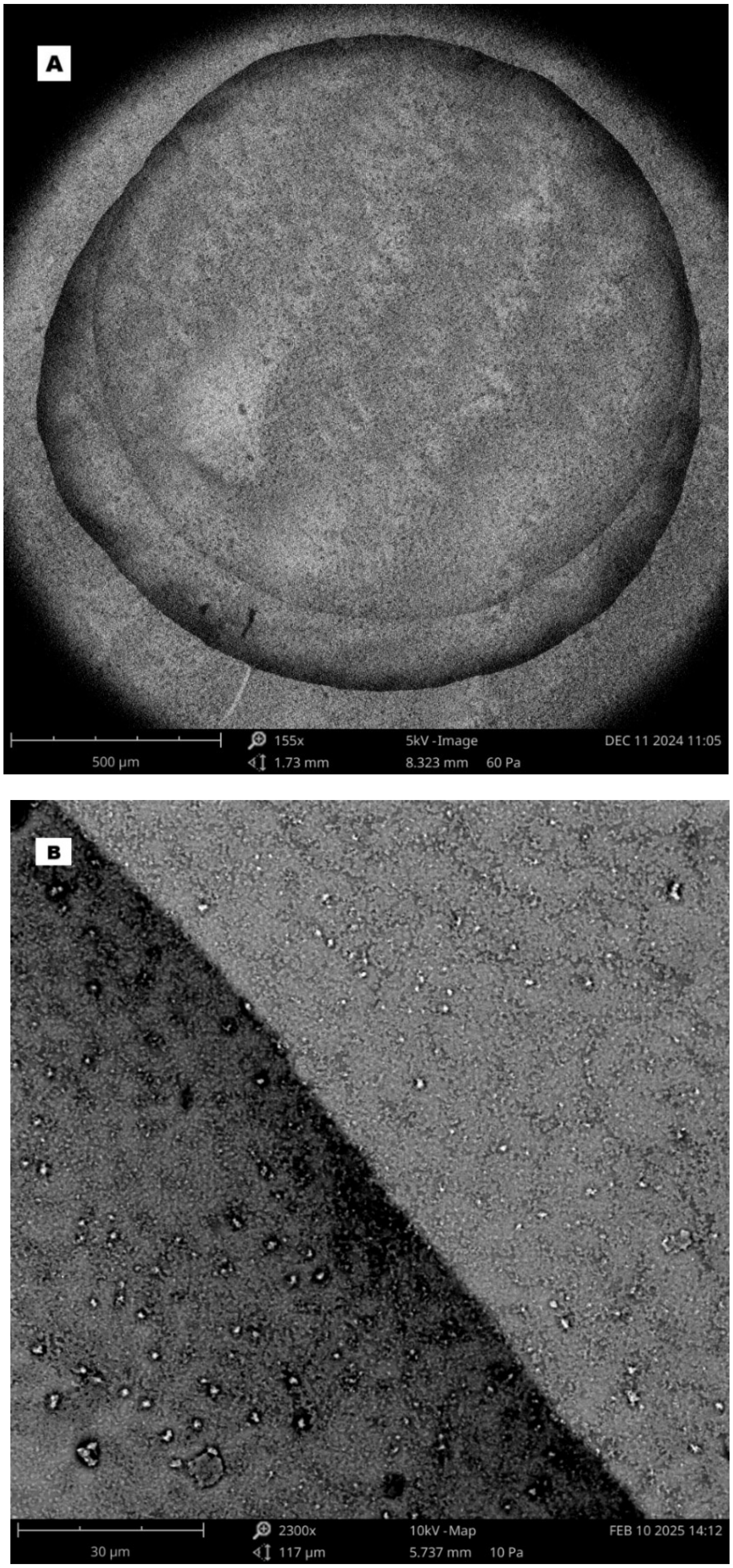

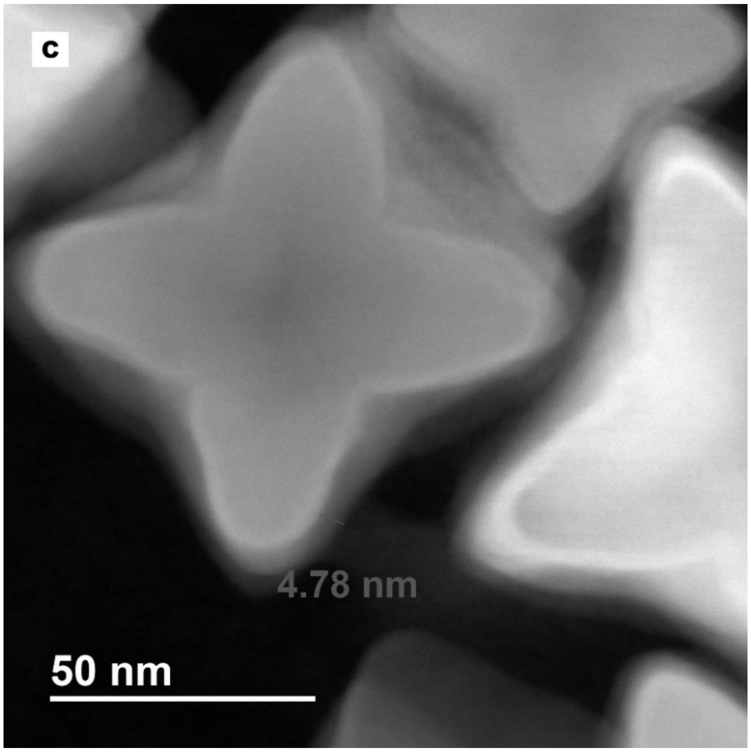
c
4.78 nm
50 nm

Figure S3. Fluorescence spectra of DA, DOPAC, and NE in five different concentrations in DI water.

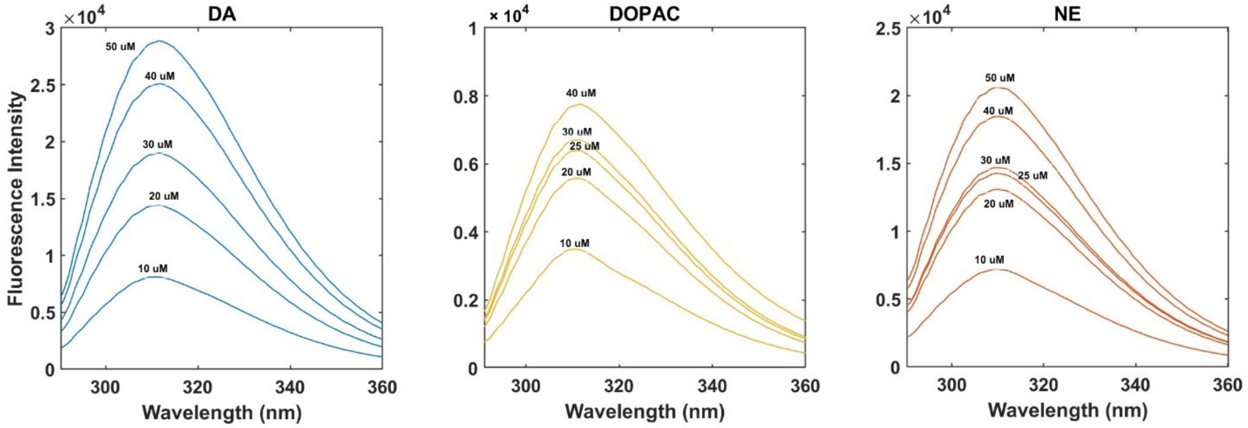

Figure S4. Absorption spectra of DA, DOPAC, and NE in five different concentrations in DI water.

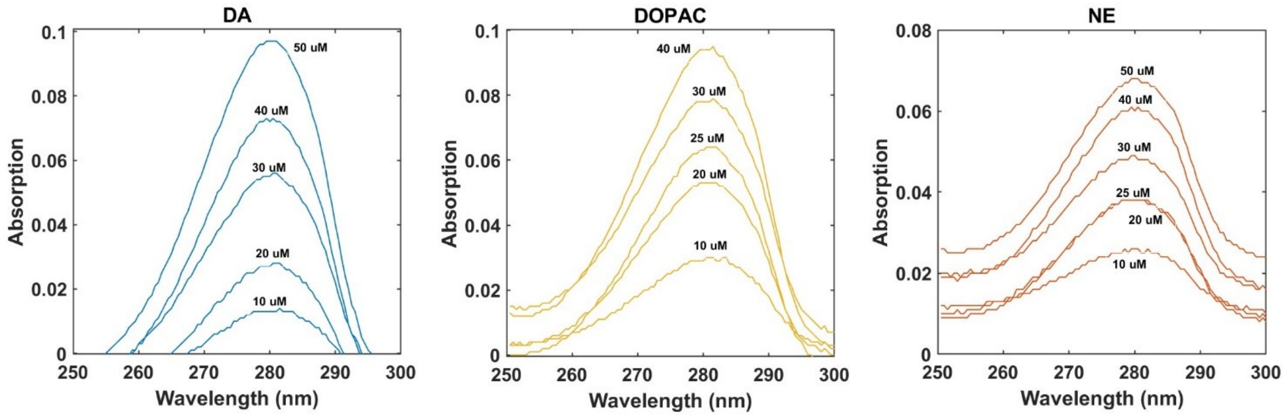

Figure S5. AFTDS spectra of DA, DOPAC, and NE on ALCNC in three different concentrations.

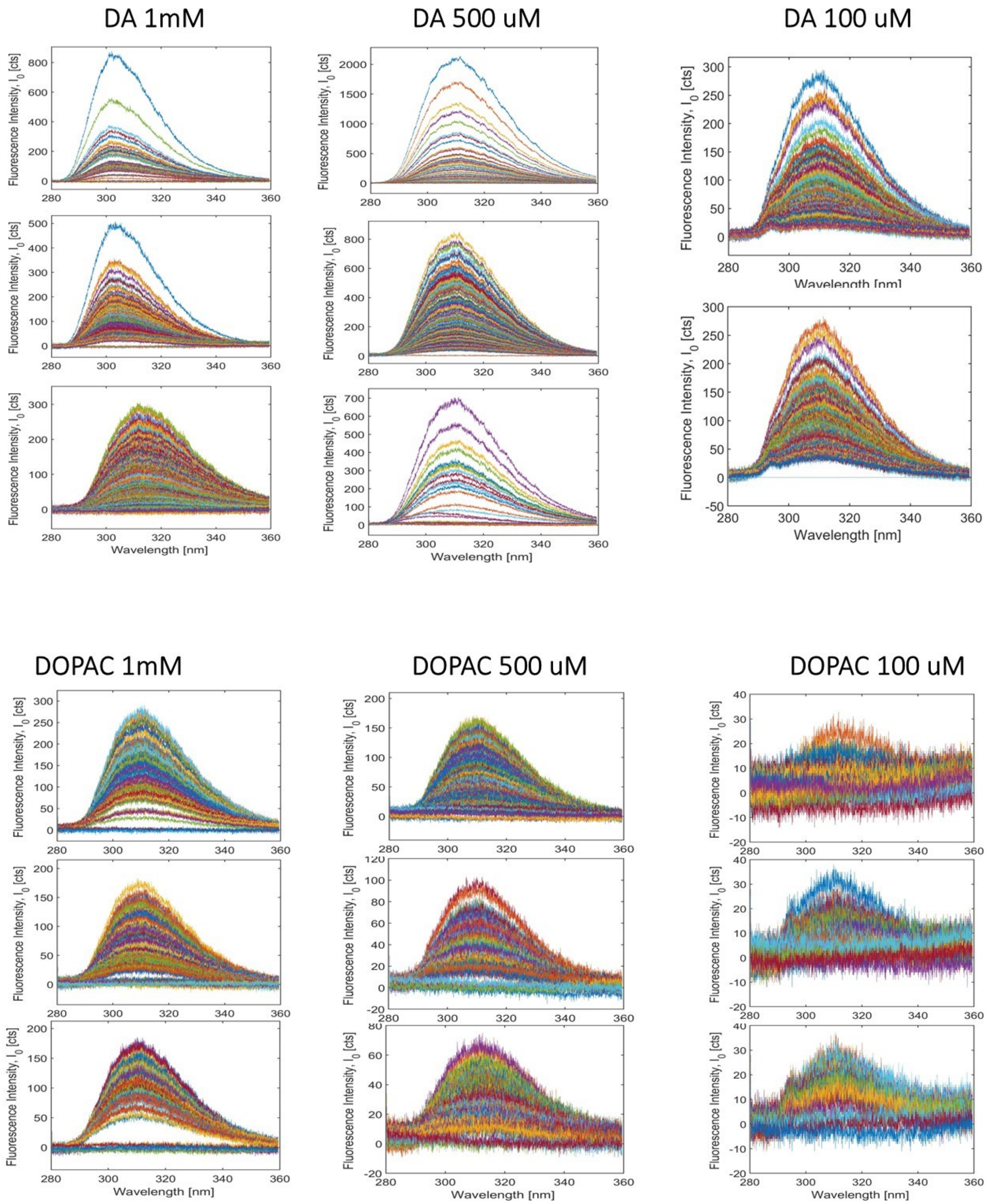

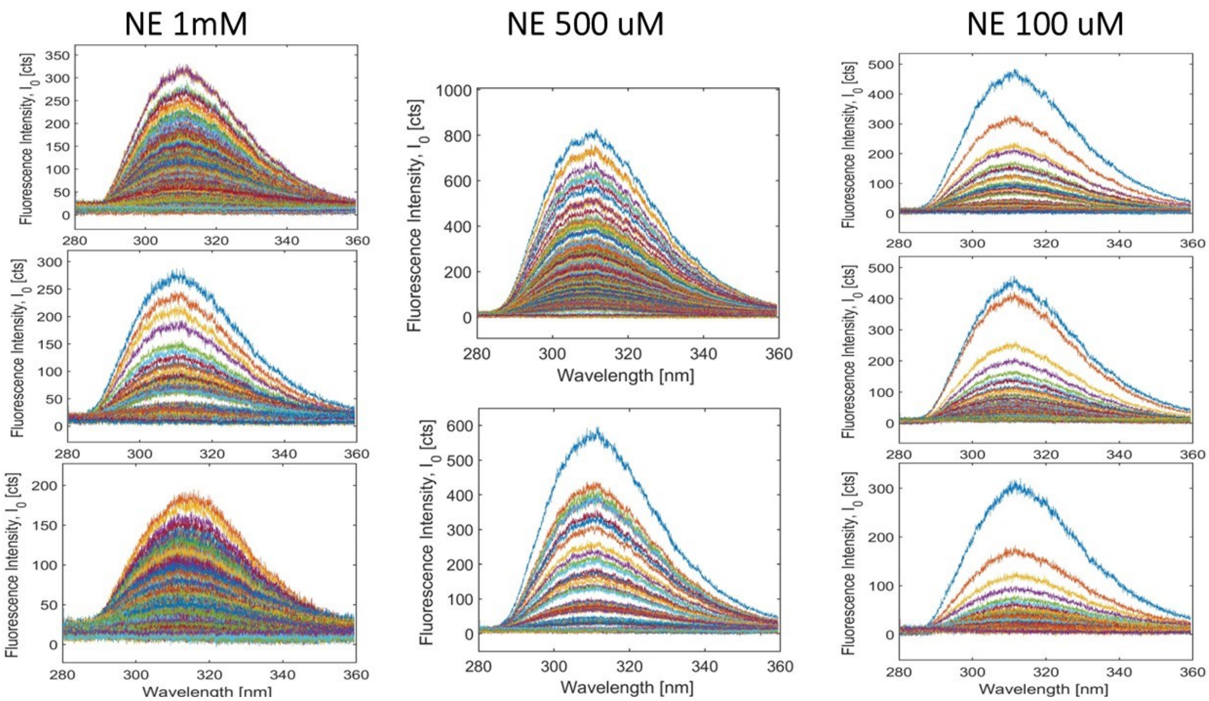

NE 1mM
NE 500 uM
NE 100 uM
Fluorescence Intensity, I0 [cts]
Wavelength [nm]

Figure S6. Confusion matrices and Table showing both percentage and sample count per classification outcome.

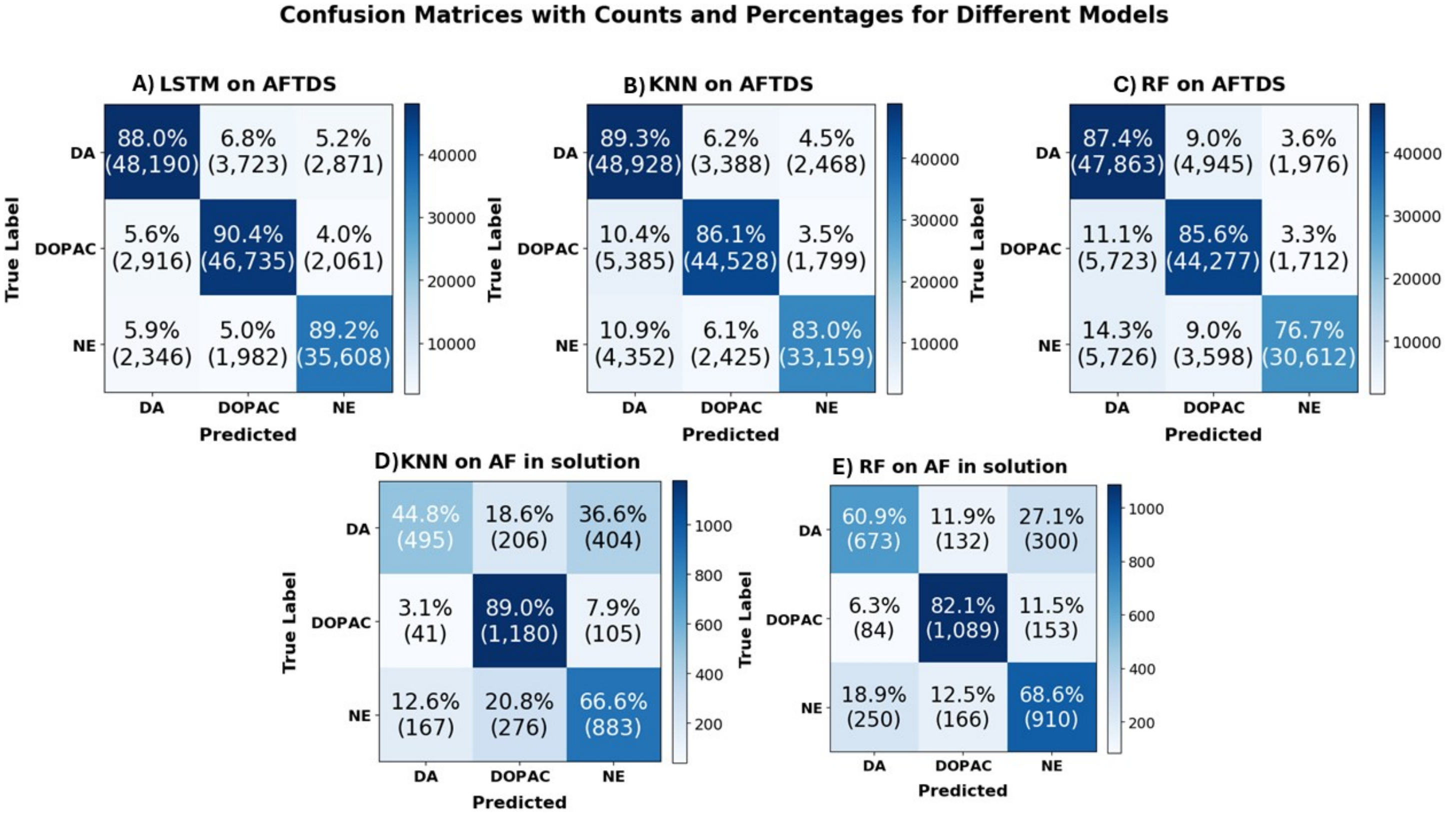

Table S3. Classification Models Performance Metrics Across Classes (DA, DOPAC, NE)

Model	Class	DA	NE	DOPAC	Weighted Avg	Macro Avg	Accuracy
**LSTM** using AFTDS of MANTs on AlCNC	**Precision**	0.90	0.88	0.89	0.89	0.89	-
	**Recall**	0.88	0.89	0.90	0.89	0.89	-
	**F1 Score**	0.89	0.88	0.90	0.89	0.89	**0.89**
	**Support**	54784	39936	51712	146432	146432	
**KNN** using AFTDS of MANTs on AlCNC	**Precision**	0.83	0.88	0.89	0.87	0.87	-
	**Recall**	0.89	0.83	0.86	0.87	0.86	-
	**F1 Score**	0.86	0.86	0.87	0.87	0.87	**0.86**
	**Support**	54784	39936	51712	146432	146432	
**RF** using AFTDS of MANTs on AlCNC	**Precision**	0.81	0.89	0.84	0.84	0.85	-
	**Recall**	0.87	0.77	0.86	0.84	0.83	-
	**F1 Score**	0.84	0.82	0.85	0.84	0.84	**0.84**
	**Support**	54784	39936	51712	146432	146432	
**KNN** using AF of MANTs in the solution	**Precision**	0.70	0.63	0.71	0.68	0.68	-
	**Recall**	0.45	0.67	0.89	0.67	0.68	-
	**F1 Score**	0.55	0.65	0.79	0.66	0.67	**0.68**
	**Support**	1105	1326	1326	3757	3757	
**RF** using AF of MANTs in the solution	**Precision**	0.67	0.67	0.78	0.71	0.71	-
	**Recall**	0.61	0.69	0.82	0.71	0.71	-
	**F1 Score**	0.55	0.65	0.79	0.66	0.67	**0.71**
	**Support**	1105	1326	1326	3757	3757	

Figure S7. Bar charts of Precision, Recall and F1 Score for DA, NE, DOPAC, Weighted Average and Macro Average.

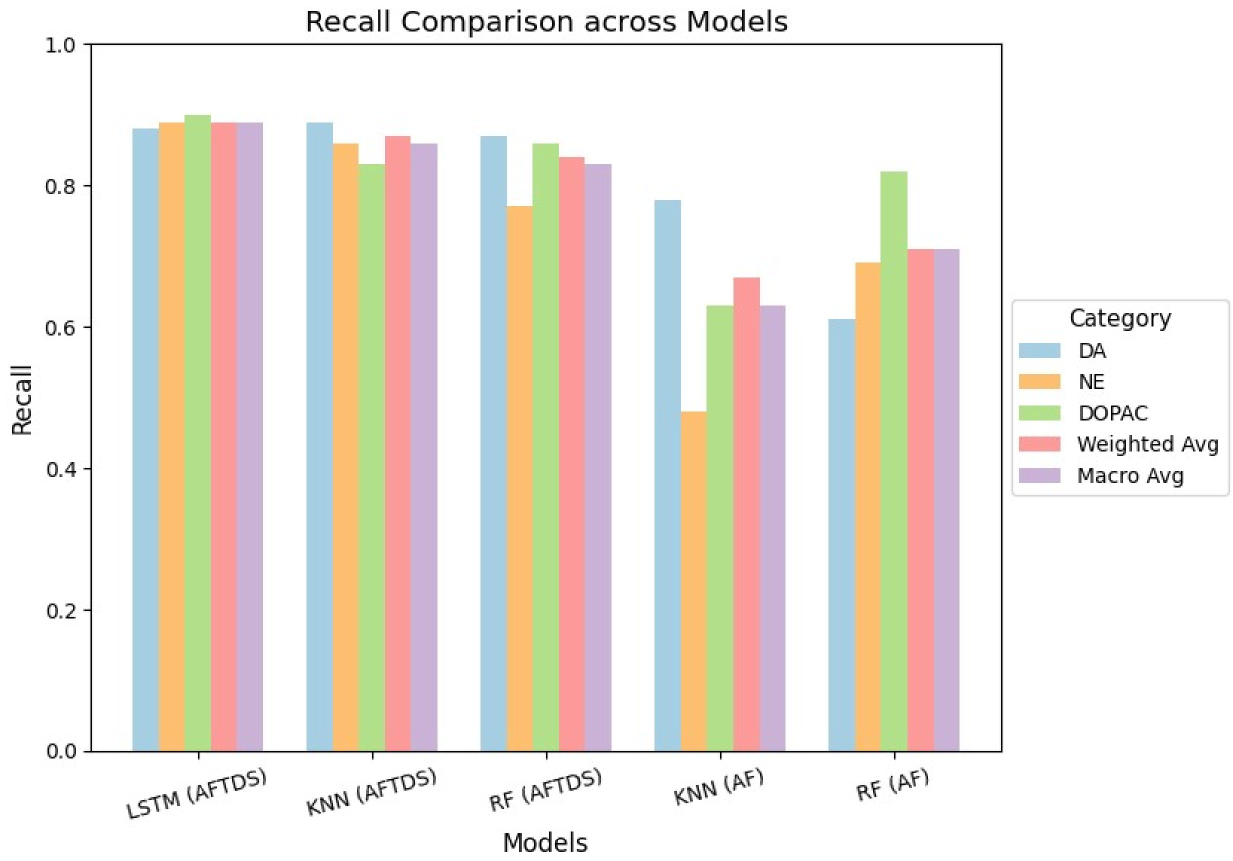

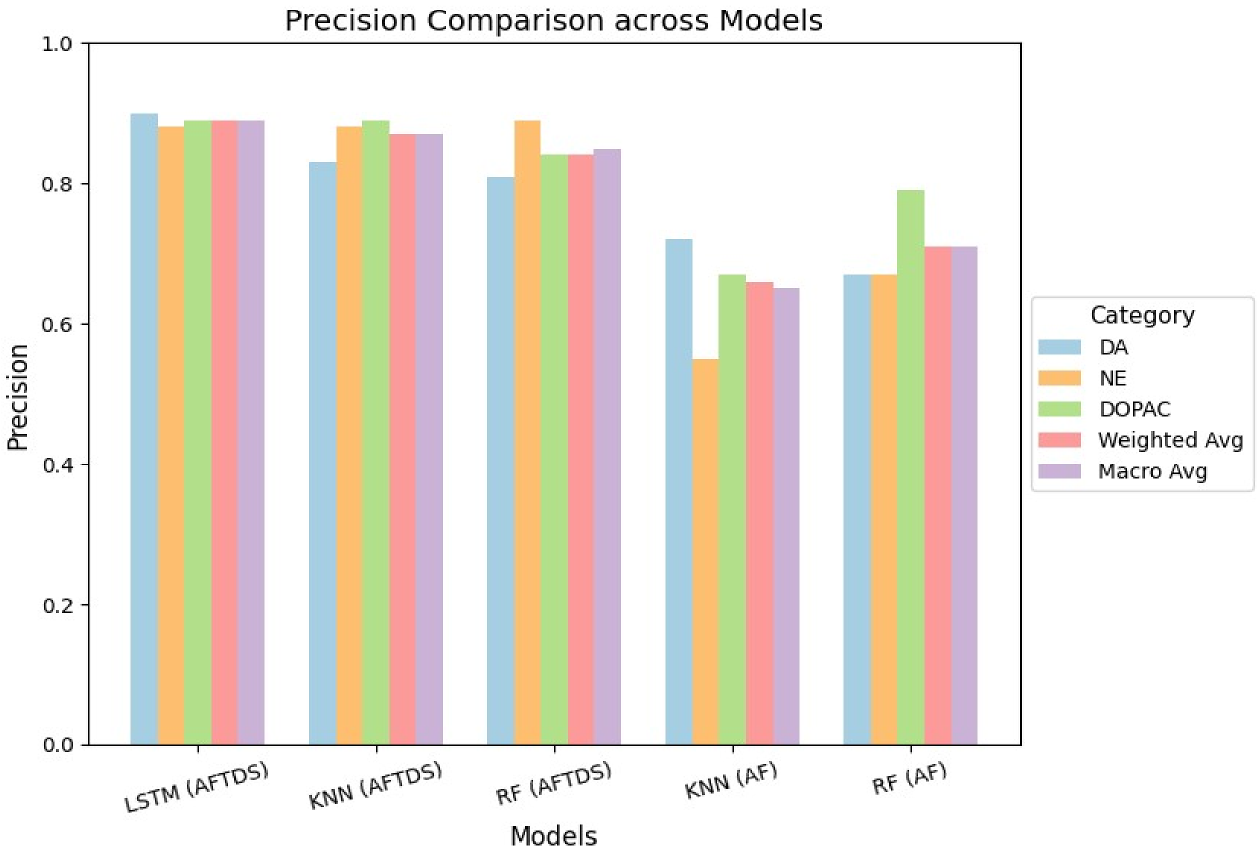
Precision Comparison across Models
1.0
0.8
0.6
0.4
0.2
0.0
Precision
LSTM (AFTDS)
KNN (AFTDS)
RF (AFTDS)
KNN (AF)
RF (AF)
Models
Category
DA
NE
DOPAC
Weighted Avg
Macro Avg

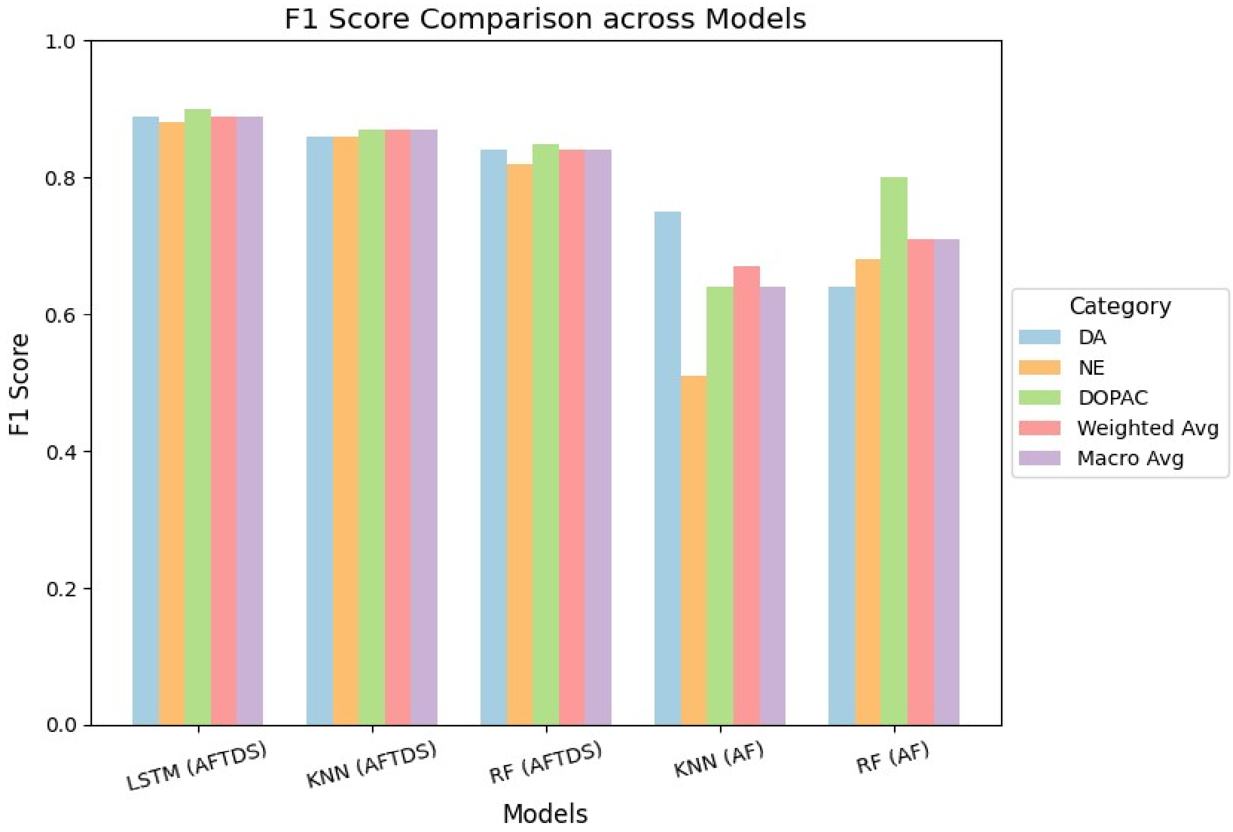
F1 Score Comparison across Models
1.0
0.8
0.6
0.4
0.2
0.0
F1 Score
LSTM (AFTDS)
KNN (AFTDS)
RF (AFTDS)
KNN (AF)
RF (AF)
Models
Category
DA
NE
DOPAC
Weighted Avg
Macro Avg

Supplementary Methods for Machine Learning Classification

Data Preprocessing

Two independent datasets were utilized in this study: (i) AFTDS data obtained from MANTs on an AlCNC substrate, and (ii) static AF spectra recorded in solution. For the AFTDS dataset, temporal autofluorescence signals corresponding to DA, DOPAC, and NE were acquired under continuous illumination. These signals were arranged into a matrix with each row representing a single time-dependent trace. Missing values were handled by shifting NaNs to the right, and shorter sequences were padded to match the length of the longest trace; additionally, traces with mixed labels were discarded and the remaining tags were standardized to "DA", "DOPAC" or "NE". Conversely, in the AF-in-solution dataset, each sample consisted of a static autofluorescence intensity recorded at specific wavelengths. The values were restructured into [wavelength, intensity] pairs for each neurotransmitter, thereby forming a feature matrix amenable to conventional machine learning approaches. In both cases, feature values were normalized to the range [0, 1] using MinMax scaling. Class labels were converted using LabelEncoder for traditional classifiers and one-hot encoding for neural network models.

Model Design and Training

AF-in-Solution Models (KNN and RF):

The AF-in-solution dataset was analyzed using classical classifiers:

- **KNN:** Following an extensive grid search, a value of k=11 was selected to optimize cross-validation performance using a 4-fold scheme.
- **RF:** A forest comprising 150 decision trees (n_estimators=150) was used, and model hyperparameters were tuned through grid search in a 4-fold cross-validation framework. Final performance was assessed on an independent 25% hold-out test set. Model performance was quantified using per-class precision, recall, F1-score, overall accuracy, and both macro and weighted averages.

Models on AFTDS (AlCNC Substrate):

The AFTDS dataset was modeled using deep learning as well as classical approaches:

- **LSTM Network:** The proposed LSTM architecture was implemented in Keras and comprised:
    - An input layer accommodating the time-series shape.
    - Three sequential LSTM layers with 200, 150, and 100 units, respectively, each followed by batch normalization to stabilize learning.
    - A fully connected (dense) layer with 50 neurons, followed by batch normalization.
    - A final SoftMax output layer for classification into the three neurotransmitter classes.

The model was trained for 50 epochs using a batch size of 64, with a 75/25 training-validation split. A learning rate scheduler was applied to reduce the learning rate upon plateauing of the validation loss.

- **KNN and RF:** In addition to the LSTM model, both KNN and RF classifiers were also applied to the AFTDS dataset. The same preprocessing pipeline was used for feature extraction, with models evaluated through 4-fold cross-validation and subsequently on a stratified 25% hold-out test set. Performance metrics, including class-wise F1-scores and confusion matrices, were reported.

Data Transformation for Time-Series Modeling

For sequence-based modeling, the AFTDS data were further preprocessed by right-aligning the time traces through shifting valid entries to the left and padding shorter sequences. Only traces exhibiting sufficient length and unambiguous class labels were retained to ensure that the dynamic spectral evolution was accurately captured by the LSTM network.

Performance Evaluation

Model performance was assessed using standard metrics: overall accuracy, per-class precision, recall, and F1-score, as well as macro and weighted averages. Confusion matrices displaying both raw counts and row-wise percentages were used to visualize classification performance and to elucidate error patterns, particularly highlighting class boundary confusion.
